# Value of prehospital assessment of spine fracture by paramedics

**DOI:** 10.1007/s00068-017-0828-0

**Published:** 2017-08-05

**Authors:** J. G. ten Brinke, W. K. Gebbink, L. Pallada, T. P. Saltzherr, M. Hogervorst, J. C. Goslings

**Affiliations:** 1 Trauma Unit, Department of Surgery, Academic Medical Center, Meibergdreef 9, 1105 AZ Amsterdam, The Netherlands; 2 Department of Surgery, Gelre Hospital Apeldoorn, Apeldoorn, The Netherlands

**Keywords:** Spinal fracture, Emergency medical services, Prehospital, Predictive value

## Abstract

**Background:**

Current guidelines state that trauma patients at risk of spine injury should undergo prehospital spine immobilization to reduce the risk of neurological deterioration. Although this approach has been accepted and implemented as a standard for decades, there is little scientific evidence to support it. Furthermore, the potential dangers and sequelae of spine immobilization have been extensively reported. The role of the paramedic in this process has not yet been examined. The aim of this study was to evaluate the accuracy of prehospital evaluations for the presence of spine fractures made by paramedics.

**Methods:**

All patients who presented with prehospital spine immobilization at our level II trauma center between January 2013 and January 2014 were prospectively included in a database. Prior to the diagnosis, paramedics recorded the probability of a spine fracture after a prehospital examination. These predictions were compared with patient outcomes. The sensitivity, specificity, positive predictive value, and negative predictive value were calculated.

**Results:**

One hundred and thirty-nine patients were included that positive predictive value was 22%, negative predictive value was 95%, sensitivity was 92%, specificity was 30%, and accuracy was 41%.

**Conclusions:**

The results of this study suggest that paramedics cannot accurately predict spinal fractures.

## Introduction

Current worldwide guidelines state that trauma patients at risk of spine injury should be immobilized by emergency medical services to reduce the risk of neurological deterioration [1–5]. The reason for these immobilization precautions is the assumption that unstable spinal injuries can deteriorate due to manipulation or movement, thereby causing secondary injury to the spinal cord [2, 6, 7]. This treatment algorithm has been accepted and implemented as the standard of care for decades, despite their being little scientific evidence to support this practice [8–12]. More than five million patients in the United States receive spinal immobilization each year [13]. The majority of blunt trauma patients do not have a spine fracture, meaning that many patients are immobilized unnecessarily. Spine immobilization can be problematic for both the patient and the paramedic: it can cause pressures sores, compromise respiration, necessitate aspiration after vomiting, raise intracranial pressure, and hamper airway management [14–17]. It is also a time-consuming intervention [17]. Development of a more selective immobilization protocol could reduce the number of immobilized patients, thereby decreasing the potential dangers and sequelae associated with unnecessary spine immobilization. Many of these selective immobilization protocols were initially designed for indications requiring radiological imaging in emergency rooms and later validated as a prehospital immobilization protocol [18–21].

Although previous research has shown that paramedics can accurately predict injury severity [22–24], the accuracy of spine fracture prediction has not yet been investigated. The purpose of this study was to answer the following question: how accurate can paramedics predict the presence of a spinal fracture?

## Methods

### Study design

This was a single institution prospective cohort study. Approval for this study was obtained from the medical ethical committee of Gelre Hospital.

### Patients and setting

All patients that presented at the emergency department of our level II trauma center with prehospital immobilization between January 2013 and January 2014 were included in a database.

### Data collection

Before radiologic imaging, paramedics recorded the probability of a spine fracture based on their own evaluation on a data collection form. They were asked to predict any spine fractures by answering ‘Yes’ or ‘No’. The paramedics also recorded the mechanism of injury (MOI). Patients were assessed according to the Advanced Trauma Life Support (ATLS) guidelines [25] and spinal imaging was performed according to Dutch guidelines [26]. The presence of a spine fracture was ruled out if computed tomography scanning was negative or if no clinical symptoms suggesting a spinal injury were detected during the 3-month follow-up period. Exclusion criteria were the absence of paramedic’s prediction and/or lack of appropriate imaging. Patients were stratified based on the prehospital prediction of a spine fracture.

### Primary outcome

Sensitivity, specificity, positive predictive value (PPV), negative predictive value (NPV), and accuracy were calculated.

### Data analysis

Data were analyzed using SPSS for Windows, Version 21.0 (SPSS Inc., Chicago, IL, USA).

## Results

**Fig. 1:**
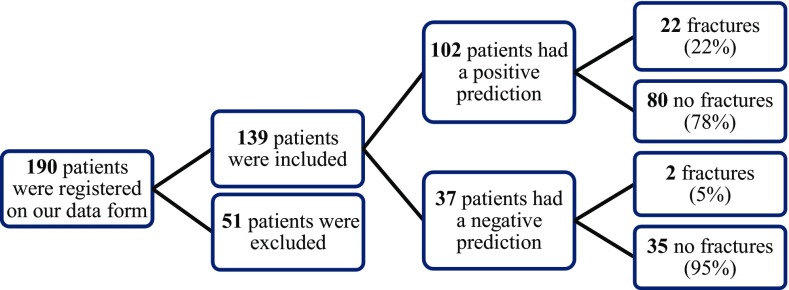
Flow diagram of patient inclusion and fracture prediction

A total of 190 patients presented with prehospital spine immobilization and 139 of these patients were included in this study (Fig. 1). The baseline characteristics and MOI are presented in Table 1. The prevalence of spine fractures was 17%. Paramedics failed to predict spine fractures in two patients (92% sensitivity and 30% specificity). The PPV was 22% and the NPV was 95%. The accuracy was 41%. The primary outcome and accuracy of this study are presented in Table 2. Twenty-two patients of the 102 were correctly predicted as having spinal fractures by the paramedics. The paramedics failed to identify spine fractures in two patients. The mechanism of injury in these patients with missed fractures was a fall from height. One of these patients had multiple spinal fractures and was given an orthesis (Table 3). Thirty-five patients were immobilized by the paramedics according to protocol, despite there being no suspicion of a spinal fracture by the paramedics (Table 2).

**Table 1:** Baseline characteristics stratified by prehospital prediction

	Positive prediction	Negative prediction
Number	102	37
Median age (IQR^a^)	41 (22–60)	23 (18–46)
Male	60%	70%
MOI^b^		
High-energy trauma	4 (41%)	17 (46%)
Isolated blunt trauma	43 (42%)	11 (30%)
Lateral impact on vertebral column	5 (5%)	3 (8%)
Other high-energy trauma	12 (12%)	6 (16%)

^a ^Inter quartile range: 25 and 75th percentile

^b^ Mechanism of injury

**Table 2:** Prehospital prediction of a spine fracture by paramedics

	Definitive diagnosis		Total
	Fracture	No fracture	
Paramedic prediction: fracture	22	80	102
Paramedic prediction: no fracture	2	35	37
Total	24	115	139

**Table 3:** Details of spine fractures in patients with a negative prediction

Patient	MOI	Fracture type	Treatment
Male, 46 years	4 m fall	T7 compression	Pain relief
Male, 69 years	2.5 m fall	T11, L2, L4 compression	Orthesis

*MOI* Mechanism of Injury

## Discussion

This study demonstrates that paramedics can predict the presence of a spinal fracture with a low degree of accuracy, as demonstrated by the accuracy of 41% found here. The decision to perform prehospital spine immobilization is currently based on PreHospital Trauma Life Support (PHTLS) criteria, which were previously reported to have an accuracy of 66% [19]. This figure was supported by a systematic review conducted in 2012 [22]. The low accuracy of the prehospital evaluations conducted by the paramedics in the current study suggests that implementation of a protocol based on paramedics predictions will not reduce the overuse of spine immobilization. The sensitivity in this study (92%) is similar to earlier findings from [19], which demonstrates that a paramedic-prediction approach will not limit sensitivity compared with current protocols. Michaleff et al. also reported a similar sensitivity to our study, although their investigation was limited to cervical spine fractures [22].

In the present study, we found that spine fractures are falsely predicted in 70% of trauma patients. Although this specificity of 30% seems low, it is in line with the results of previous studies that have looked at current guidelines [19, 22, 27]. Furthermore, the NPV of paramedic spine fracture evaluations reported here (95%) is the same as that reported in the current protocol for spine immobilization [1, 19].

Paramedics failed to predict spine fractures in two patients (5%), mainly because the symptoms were mild at initial presentation and a painful distracting injury was present. At the 3-month follow-up, only one of these patients still had symptoms, while the other had fully recovered. None of these patients sustained spinal cord injury.

One limitation of this study was the high number of exclusions. The main reason for exclusion was the absence of a completed data form. Another limitation of our study is that we did not consider how many years of experience the paramedics had; it is possible that more experienced paramedics can more accurately predict spinal fractures in trauma patients. In addition, patients did not receive a CT scan of the whole spine, only of the segments that had an indication for this type of imaging. Some spine fractures may, therefore, have been missed, although the clinical relevance of a missed fracture is questionable. The fact that this study was performed in a level II trauma center means that patients with extreme severe trauma and a high chance of positive prediction are excluded. The high prevalence of spine fractures in this study (17%) relative to the prevalence found in the literature (3−5%) [28] could be caused by how we selected the patients in our study. We selected all patients that had undergone prehospital immobilization according to our national protocol. However, recent numbers of spinal fractures are increasing due to osteoporosis and the increased use of computed tomography scans [29].

This study could be added to by further investigating how accurately paramedics can predict the location of spine fractures and whether cervical, thoracic, or lumbar fractures are easiest to predict. Future studies should analyze a larger number of paramedics and take the fracture location and years of experience into account.

## Conclusion

Paramedics cannot accurately predict spinal fractures based on the trauma mechanism and clinical symptoms.
